# Draft genome sequence of the halophilic *Halobacillus mangrovi* KTB 131 isolated from Topan salt of the Jeon-nam in Korea

**DOI:** 10.1016/j.gdata.2017.07.010

**Published:** 2017-07-23

**Authors:** Mingyeong Woo, Sun-Hee Park, Kyounghee Park, Min-Kyu Park, Ji-Yeon Kim, Han-Seung Lee, Jae Hak Sohn, Dong-Woo Lee, Gaewon Nam, Kee-Sun Shin, Sang-Jae Lee

**Affiliations:** a Major in Food Biotechnology and The Research Center for Extremophiles & Marine Microbiology, Silla University, Busan 46958, South Korea; b School of Applied Bioscience, Kyungpook National University, Daegu 41566, South Korea; c Department of Bio-cosmetic Science, Seowon University, Cheongju 28674, South Korea; d Industrial Bio-materials Research Center, Korea Research Institute of Bioscience and Biotechnology (KRIBB), Daejeon 34141, South Korea

## Abstract

The draft genome sequence of the halophilic bacterium *Halobacillus mangrovi* KTB 131, isolated from Topan salt of the Jeon-nam in Korea, was established. The genome comprises 4,151,649 bp, with a G + C content of 41.6%. The strain displays a high number of genes responsible for secondary metabolite biosynthesis, transport, and catabolism compared to other *Halobacillus* bacterial genus members. Numerous genes responsible for various transport systems, solute accumulation, and aromatic/sulfur decomposition were detected. The first genomic analysis encourages further research on comparative genomics and potential biotechnological applications. The whole draft genome sequence of *Halobacillus mangrovi* KTB 131 is now available (Bioproject PRJNA380285).

**Table:** 

Specifications	
Organism/cell line/tissue	*Halobacillus mangrovi* KTB 131
Sequencer or array type	PacBio RS II
Data format	Analyzed
Experimental factors	Assembled and annotated whole genome
Experimental features	Isolated genomic DNA from strin and 16S rRNA gene sequence
Consent	N/A
Sample source location	Topan salts of the Shin-Ahn tae-pyung saltern, Korea

## 1 Direct link to deposited data

https://www.ncbi.nlm.nih.gov/nuccore/CP020772.1.

## 2 Introduction

The genus *Halobacillus* was created by Spring et al. with the description of two novel species, *Halobacillus litoralis* and *Halobacillus trueperi*, and represents a large group of halophilic aerobic bacteria (Gram-positive, rod-shaped, heterotrophic, endospore-producing) belonging to the family *Bacillaceae* [1]. To date, 20 species have been described within the genus *Halobacillus*, which are widely distributed among diverse natural saline environments such as marine salterns, salt lakes, saline soils, salt-fermented foods, and salt-preserved food products [2], [3]. Hence, more investigations at the genomic level are required to improve our understanding of its ecology, genetics, and potential biotechnological applications. The *Halobacillus mangrovi* KTB 131 strain was isolated from the Topan salt of Shin-Ahn tae-pyung saltern in Korea. Topan defines a marine solar saltern's floor turned into red-clay using a Korean traditional method. To date, the whole-genome analysis of *Halobacillus mangrovi* had not been reported. To fill this gap, *Halobacillus mangrovi* KTB 131 was chosen to perform genome sequencing.

## 3 Materials and methods

Genome sequencing was accomplished using a single molecule real-time (SMRT) sequencing platform on the PacBio RS II (Pacific Biosciences, Menlo Park, CA) [4]. Genomic DNA was isolated using a standard genomic DNA isolation kit (Promega, USA). The whole genome sequencing of strain SAH-A6 was accomplished using single SMRT cell with a single 180-min movie (Pacific Biosciences) with P6C4 chemistry [5]. The open reading frames of the assembled genome were predicted and annotated using the hierarchical genome-assembly process (HGAP) [6] protocol RS HGAP Assembly 2 in SMRT analysis version 2.3.0 (Pacific Biosciences; https://github.com/PacificBiosciences/SMRT-Analysis), IMG-ER [7], NCBI COG function [8], Pfam information [9], and EzTaxon [10] database. The rRNA and tRNA genes were identified using RNAmmer 1.2 [11] and tRNA scan-SE 1.23 [12], respectively. The whole genome sequence of SAH-A6 was annotated using the Rapid Annotation System Technology (RAST) server. The pie chart showed the counts for each subsystem feature as well as the subsystem coverage.

## 4 Data description

**Fig. 1:**
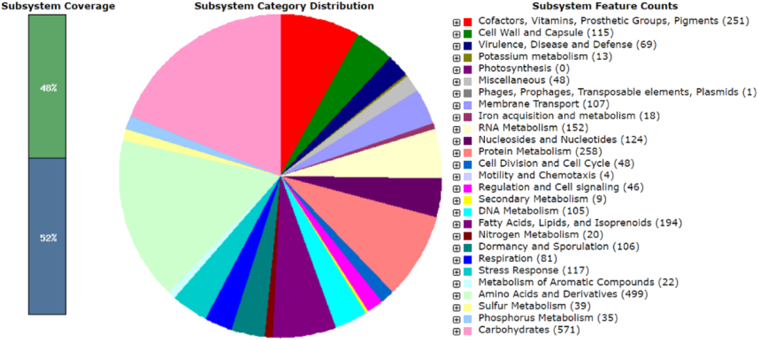
The subsystem category distribution statistics for *Halobacillus mangrovi* KTB 131. The whole genome sequence of KTB 131 was annotated using the Rapid Annotation System Technology (RAST) server. The pie chart showed the count of each subsystem feature and the subsystem coverage.

Moderately halophilic KTB 131 strain grows at NaCl concentrations ranging between 5 and 20% (w/v), with optimum growth obtained at 10% (w/v). Growth occurs at temperatures of 10–45 °C and pH 7.0–9.0. The KTB 131 strain showed the ability to hydrolyze skim milk, starch, and tween 80. A phylogenetic tree was built based on a neighbor joining tree obtained from the alignment of the 16S rRNA gene sequences (~ 1400 bp), showing the relationship between *Halobacillus* sp. genomes available for KTB 131 using MEGA 6 (Supplementary Fig. 1). The draft genome sequence of *Halobacillus mangrovi* KTB 131, isolated from Topan salts of the Shin-Ahn tae-pyung saltern, Korea, was determined. The assembled genome comprises 4,151,649 bp, with a G + C content of 41.6%. Strain KTB 131 displays a G + C content similar to those observed in other *Halobacillus* sp. (Table 1). The strain possesses a high number of genes that are responsible for secondary metabolites biosynthesis, transport, and catabolism compared to other bacteria from the *Halobacillus* genus. In addition, strain KTB 131 uses universal strategies toward enabling extreme adaptation, as indicated by its genome. Numerous genes responsible for various transport systems, solute accumulation, and aromatic/sulfur decomposition were detected. Additionally, as shown in Fig. 1, this strain displays many genes involved in the Serine-glyoxylate cycle, sporulation gene orphans, the glycolipid and glycerophospholipid metabolisms, fatty acid biosynthesis FASII, maltose and maltodextrin utilization, ribosomal LSU production, and modification of tRNA involved in peptidoglycan synthesis. The results obtained from the subsystem category distribution statistical analysis for *Halobacillus mangrovi* KTB 131 are shown in Fig. 1.

**Table 1:** Comparison of the genomic feature of *Halobacillus mangrovi* KTB 131 strain with various halophilic *Halobacillus* strains. The information of the reference genomes was obtained from NCBI data base.

Organism	BioProject	Resource	Genome size	Contigs	G + C (%)	r + tRNA
*H. mangrovi* KTB 131^a^	PRJNA380285	Jeon-nam, Korea	4,151,649	1	41.6	11 + 42
*H. salinus* HSL-3	PRJNA356196	East sea, Korea	3,766,720	4	44.3	20 + 69
*H. alkalipholus* Fp5	PRJNA323265	Fuente de Piedra, Spain	4,092,530	103	41.6	18 + 56
*H. aidingenisis* CGMCC 1.3703	PRJNA329899	Xin-Jiang, China	4,191,840	53	43.5	20 + 47
*H. Kuroshimensis* DSM 18393	PRJNA188908	Japan	3,845,570	16	47.0	13 + 55

a This study.

The following are the supplementary data related to this article.

Supplementary Fig. 1 Phylogenetic tree constructed using the neighbor-joining method based on 16SrRNA gene sequences, showing the taxonomic position of strain KTB 131 in the genus *Halobacillus*. The information of the reference genomes was obtained from EzTaxon data base. Supplementary Fig. 1

## Verification and authentication

The whole draft genomic sequence of *Halobacillus mangrovi* KTB 131 (Bioproject PRJNA380285) has been deposited at NCBI GenBank database under accession numbers CP020772. This strain is available from Korean Collection for Type Cultures (KCTC) with the accession number KCTC 33901.
