# Supplementary material for: Draft genome sequence of the halophilic Halobacillus mangrovi KTB 131 isolated from Topan salt of the Jeon-nam in Korea
Source: Genom Data. 2017 Jul 23;14:18–20. doi: 10.1016/j.gdata.2017.07.010 (PMC5554927; doi:10.1016/j.gdata.2017.07.010)
Supplement: Supplementary Fig. 1 — Phylogenetic tree constructed using the neighbor-joining method based on 16SrRNA gene sequences, showing the taxonomic position of strain KTB 131 in the genus Halobacillus. The information of the reference genomes was obtained from EzTaxon data base. [file mmc1.pdf]

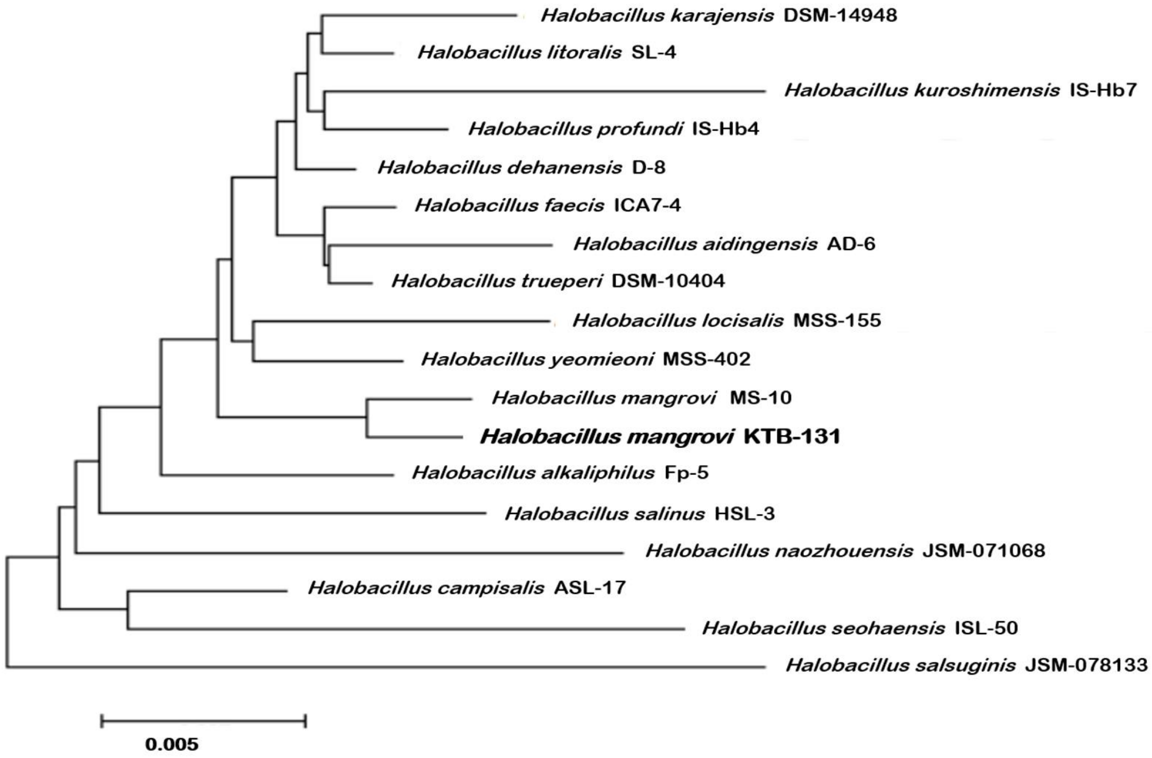

Supplementary Fig. 1. Phylogenetic tree constructed using the neighbor-joining method based on 16SrRNA gene sequences, showing the taxonomic position of strain KTB 131 in the genus *Halobacillus*. The information of the reference genomes was obtained from EzTaxon data base.
